# Identification and Characterization of SQUAMOSA Promoter Binding Protein-like Transcription Factor Family Members in *Zanthoxylum bungeanum* and Their Expression Profiles in Response to Abiotic Stresses

**DOI:** 10.3390/plants14040520

**Published:** 2025-02-08

**Authors:** Shengshu Wang, Weiming Hu, Xueli Zhang, Yulin Liu, Fen Liu

**Affiliations:** 1 College of Forestry, Northwest A&F University, Yangling 712100, China; wss24616@163.com (S.W.); zxl980505@nwafu.edu.cn (X.Z.); 2 Lushan Botanical Garden, Jiangxi Province and Chinese Academy of Sciences, Jiujiang 332900, China; huwm@lsbg.cn

**Keywords:** *SQUAMOSA promoter binding protein-like (SPL)*, *Zanthoxylum bungeanum*, transcription factors, abiotic stress, gene expression

## Abstract

Plant-specific transcription factors known as SQUAMOSA promoter binding protein-like (*SPL*) genes are essential for development, growth, and abiotic stress responses. While the *SPL* gene family has been extensively studied in various plant species, a systematic characterization in *Zanthoxylum bungeanum* (*Zb*) is lacking. This study used transcriptomic and bioinformatics data to conduct a thorough genomic identification and expression investigation of the *ZbSPL* gene family. Eight subfamilies including 73 *ZbSPL* members were identified, most of which are predicted to be localized in the nucleus. Ka/Ks ratio analysis indicates that most *ZbSPL* genes have undergone purifying selection. According to evolutionary research, segmental duplication is a major factor in the amplification of the *ZbSPL* gene family. Gene structures, conserved motifs, and domains were found to be highly conserved among paralogs. *Cis*-element research revealed that *ZbSPLs* may be implicated in hormone and abiotic stress responses. Codon usage pattern analysis showed that the *ZbSPL* gene family was more inclined to A/T base endings; the higher the A/T content, the stronger the preference of the codons; and the use pattern was mainly affected by natural selection. Additionally, 36 *ZbSPLs* were found to be potential targets of miR156. RNA-seq demonstrated that *SPL* genes in *Zb* are differentially expressed in response to distinct abiotic stressors. Two *ZbSPL* genes (*ZbSPL10* and *ZbSPL17*) were implicated in the response to salt stress, while four *ZbSPL* genes (*ZbSPL06*, *ZbSPL43*, *ZbSPL60*, and *ZbSPL61*) showed response to drought stress, based on a qRT-PCR investigation of the *ZbSPL* genes under various abiotic stress conditions. This study will help us gain a deeper understanding of the functions of *ZbSPLs* and lay a genetic foundation for future breeding of high-quality, highly abiotic resistant varieties of *Z. bungeanum*.

## 1. Introduction

Transcription factors (TFs) play a crucial role in regulating gene expression by either activating or repressing target genes [1,2]. These DNA-binding proteins are essential for controlling a wide range of plant biological processes, including growth, development, and stress responses [3,4]. The SQUAMOSA Promoter Binding Protein (SPL) family is a group of plant-specific transcription factors. First discovered in *Antirrhinum majus*, *SBP1* and *SBP2* were found to have a significant impact on the early stages of flower development [5]. They used the method of screening the cDNA expression library to demonstrate that the expression levels of *SBP1* and *SBP2* were closely associated with the initiation and progression of floral organogenesis. Subsequent research [6] has further explored the molecular mechanisms underlying the function of *SPLs* in flower development and other plant physiological processes. The highly conserved 76-amino acid SBP domain of the SPL proteins contains a C-terminal nuclear localization signal (NLS) and two zinc fingers (found at the Zn-1 and Zn-2 zinc binding sites) that are crucial for DNA binding and nuclear localization [7,8,9,10]. In *Arabidopsis thaliana* (*At*), 16 *SPL* family members have been identified and classified into eight subgroups [11]. As more plant genomes have been sequenced, additional *SPL* family members have been identified and studied across various species, including rice (*Oryza sativa*) [12], tobacco (*Nicotiana tabacum*) [13], citrus (*Citrus clementina*) [14], poplar (*Populus euphratica*) [15], barley (*Hordeum vulgare*) [16], and wheat (*Triticum aestivum*) [17]. These investigations have revealed that *SPL* genes are involved in a wide range of plant growth processes, including vegetative growth [18,19], flowering and fruit development [20,21], and hormone regulation [22,23]. More recent investigations have also highlighted the crucial role that *SPL* genes play in regulating abiotic stress tolerance in a range of plant species. *SPL1* and *SPL12* in *At*, for example, are known to provide heat tolerance throughout the reproductive stage, and their overexpression boosts seed yield and heat resistance of the inflorescence [24]. In rice, *OsSPL10* controls drought tolerance through the regulating the expression of *OsNAC2*, which impacts stomatal movement and reactive oxygen species (ROS) production [25]. It also influences soil metabolites to promote salt stress resistance [26].

Additionally, miR156 negatively regulates many *SPL* family members through mRNA cleavage or post-transcriptional repression [27], which can either improve or impair a plant’s response to abiotic stress. *TcmiR156*, for example, may play an important role in salt stress response by negatively controlling *TcSPLs* [28]. *MdWRKY100* is upregulated by the miR156/*SPL* module, which also improves salt resistance in apples [29]. Growing evidence suggests that the miR156/*SPL* module is a crucial mediator in balancing plant responses to abiotic stress and developmental processes.

*Zanthoxylum bungeanum* (*Zb*) is widely distributed and possesses significant economic and medicinal value. Nevertheless, no prior research has been conducted on the *SPL* gene family in *Zb*. In this investigation, *ZbSPL* genes in the *Zb* genome were identified and characterized. Comprehensive examinations of their gene structures, chromosomal locations, phylogenetic relationships, synteny, codon preference, miR156 binding sites, and *cis*-regulatory elements were also conducted. Additionally, we investigated the expression patterns of *ZbSPL* genes under various abiotic stress conditions, providing insights into their biological functions. The results from this thorough examination of the *ZbSPL* gene family establish a foundation for further studies into the functional differentiation and possible uses of these genes.

## 2. Results

### 2.1. Identification of ZbSPL Gene Family and Analysis of Its Physicochemical Properties

Our results demonstrated that the *Zb* genome harbored a total of 73 *SPL* genes. These genes were categorized as *ZbSPL01* through *ZbSPL73* based on their chromosomal locations (Table S1). In terms of their amino acid sequence, the ZbSPL proteins range in length from 126 aa (ZbSPL47/48) to 2084 aa (ZbSPL59), and their molecular weights range from 14.46 kDa (ZbSPL48) to 230.75 kDa (ZbSPL59). The ZbSPL proteins are hydrophilic, as evidenced by their negative average hydrophilicity values. Only ZbSPL01, ZbSPL02, and ZbSPL25 are anticipated to be found in the plasma membrane, according to subcellular localization predictions, which showed that the bulk of the ZbSPL proteins are located in the nucleus (Table S2).

### 2.2. Chromosome Localization and Gene Replication

**Figure 1:**
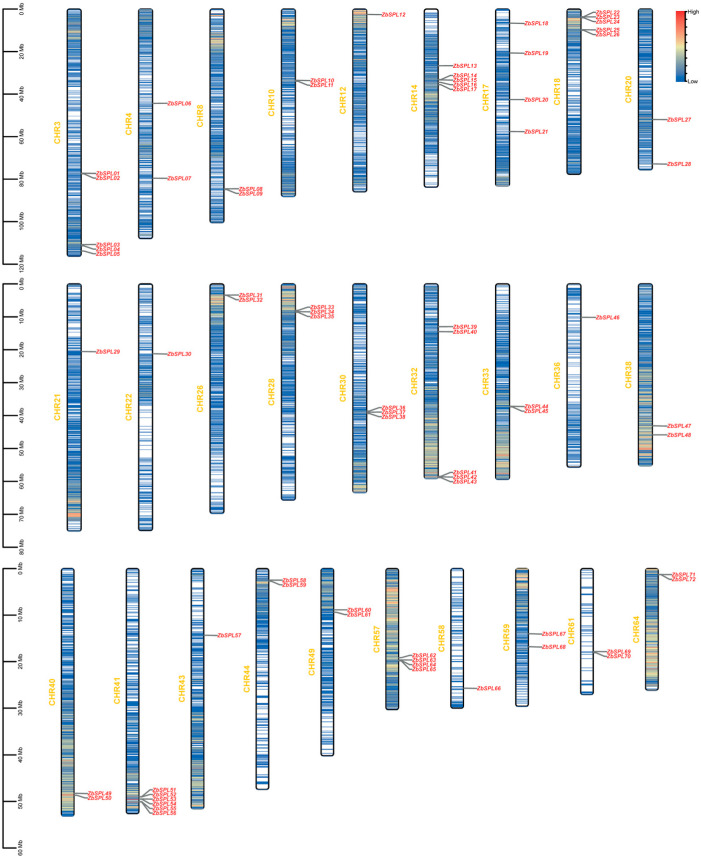
The distribution of the *ZbSPL* genes on the chromosomes of *Zb*. The scale indicates chromosome length. The variation in the shade of the center color indicates gene density.

**Figure 2:**
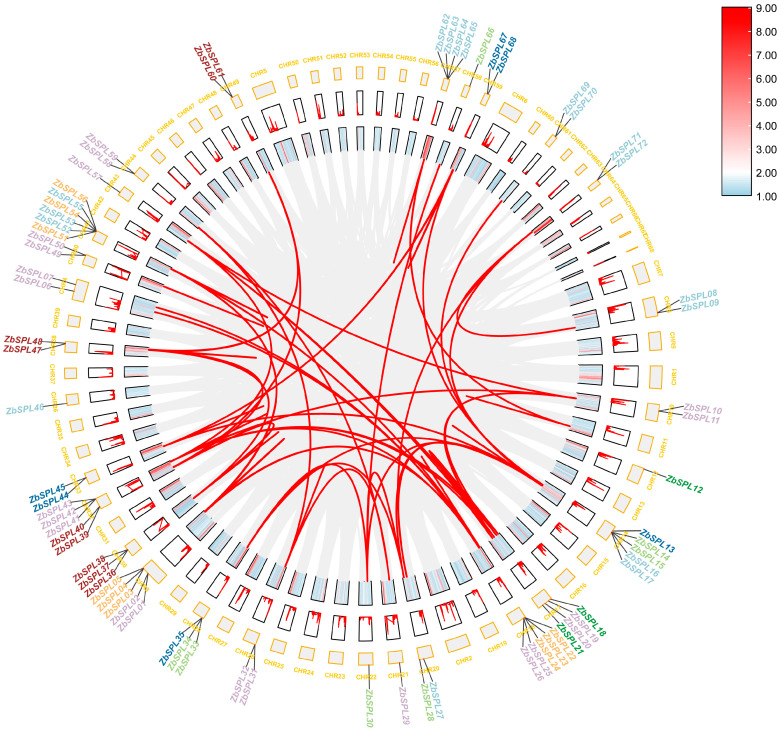
Synteny analysis of *ZbSPLs* within *Zb* genome. Red lines represent collinearity of *ZbSPL* gene; gray lines represent collinearity of entire *Zb* genome.

According to the genomic data, there are 72 *ZbSPLs* in *Z. bungeanum* (Figure 1), with one not dispersed among the chromosomes. Chromosome 41 contains the greatest number of *ZbSPL* genes (6), as shown in Figure 1. On the other hand, only one *ZbSPL* gene was found on each chromosome for chromosomes 12, 21, 22, 36, and 43. To gain insights into the mechanisms of gene amplification in the *ZbSPL* gene family, we analyzed repetitive events in the *Zb* genome. Only 107 pairs of segmental duplications and 2 pairs of tandem duplications were found among these 73 *ZbSPL* genes, as shown in Figure 2 and Table S3, indicating that segmental duplications are the primary mechanism for expansion of the *ZbSPL* gene family. Only the 12th and 36th chromosomes did exhibit any duplications, whereas the *ZbSPL* genes on 27 other chromosomes were implicated in these duplication events. Therefore, the *ZbSPL* gene family has experienced purifying selection throughout its evolutionary history, as evidenced by the fact that all gene pairs implicated in duplications have a Ka/Ks (non-synonymous/synonymous) ratio < 1 (range: 0.08–0.89).

**Figure 3:**
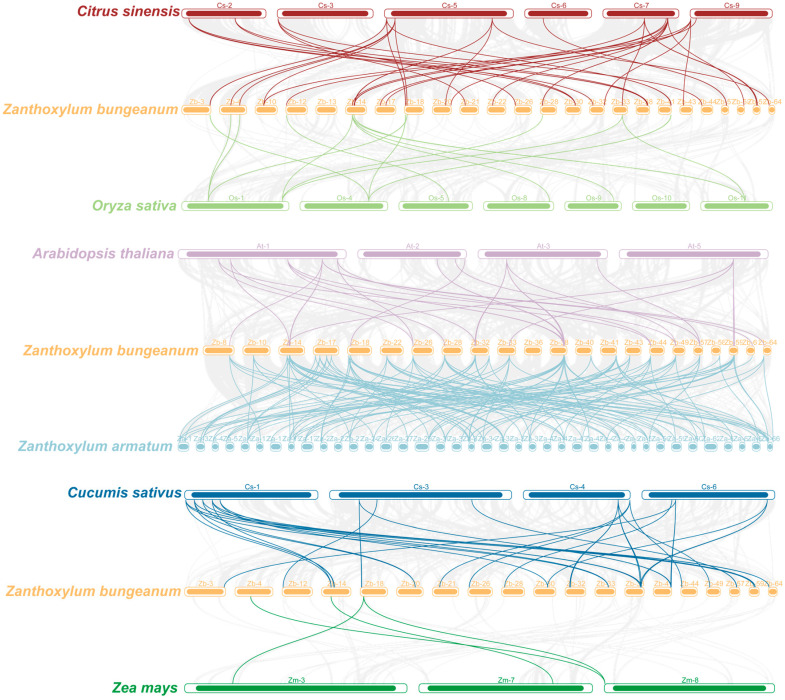
An analysis of syntenic relationships between *ZbSPL* genes in *Zb*, *Csi*, *Os*, *At*, *Za*, *Csa*, and *Zm*. Syntenic *SPL* gene pairs are highlighted by lines with colors corresponding to the color of the genome *Zb* is being compared to. The gray lines represent collinearity across the entire genome between different species and *Zb*.

To better understand the origin and evolutionary relationships of *ZbSPL* genes, we analyzed the synteny relationships between *SPL* genes in *Zb* and those in *At*, *Zanthoxylum armatum* (*Za*), *O. sativa* (*Os*), *C. sinensis* (*Csi*), *Cucumis sativus* (*Csa*), and *Z. mays* (*Zm*). As shown in Figure 3, we identified 46, 271, 20, 64, 46, and 7 syntenic *SPL* gene pairs in *At*, *Zb*, *Os*, *Csi*, *Csa*, and *Zm*, respectively. There is not a one-to-one correspondence between all *SPL* genes in *Zb* and *Za*, but rather a many-to-many relationship, indicating that genome rearrangement and reshuffling of *SPL* orthologs occurred after the divergence of *Zb* and *Za*. The *SPL* genes of *Csi* and *Zb* are more evolutionarily conserved and share a closer phylogenetic link between species. This high similarity suggests they may have developed from a common ancestor shared by several plants.

### 2.3. Analysis of Conserved Motifs, Conserved Binding Domains, and Gene Structure

The presence of shared motifs among different proteins was determined based on the 20 conserved motifs identified within the ZbSPL family. As shown in Figure S1b, the number of conserved motifs per protein ranges from three to sixteen. Subfamily II contains the highest number of conserved motifs (16), whereas subfamilies VI and VII have the fewest (3). Within each subfamily (Figure S1a), most ZbSPL members share a common motif composition. For instance, all members of subfamily II, which have the most conserved motifs, share 15 common motifs. Subfamily I members have nine common motifs, while subfamilies V and VIII each have four common motifs, and the remaining three subfamilies have three common motifs each. Motif numbers and arrangements vary between subfamilies. Conserved motifs 1, 3, and 4 are present in all ZbSPL proteins, arranged in the specific order of motif 3, followed by motif 1, and then by motif 4.

Analysis of the conserved domains revealed that all identified ZbSPL proteins possess an SBP domain (Figures S1c and S2), with most subfamily II members containing an additional Ankyrin repeat domain-containing protein domain (ANKYR). Further sequence alignment showed that the SBP domain of the ZbSPL proteins is approximately 76 amino acids in length and includes two conserved zinc-binding sites (Zn1 and Zn2) and an NLS. In subfamily IV, the His residue at the Zn1 site is replaced by a Cys residue, resulting in a characteristic Cys-Cys-Cys-Cys sequence for family members such as ZbSPL12, ZbSPL18, and ZbSPL21, in contrast to the Cys-Cys-Cys-His sequence found in the other subfamilies. Regarding the Zn2 site, the remaining proteins exhibit the Cys-Cys-His-Cys motif, with the exception of ZbSPL03, ZbSPL04, and ZbSPL05, where the His residue is substituted by Gln. Additionally, ZbSPL49 and ZbSPL50 lack an NLS. Further development of the protein phylogenetic tree and mapping of the intron/exon structure of the ZbSPL gene family will allow for a more thorough assessment of the structural traits of related genes. The number of introns in the 73 *ZbSPL* genes ranges from one to eleven (Figure S1d). Subgroup II contains the highest average number of introns, while subgroup VI contains the lowest. The majority of *ZbSPL* genes in the same subgroup have comparable gene architectures, especially regarding exon length and intron count.

### 2.4. Phylogenetic Analysis

**Figure 4:**
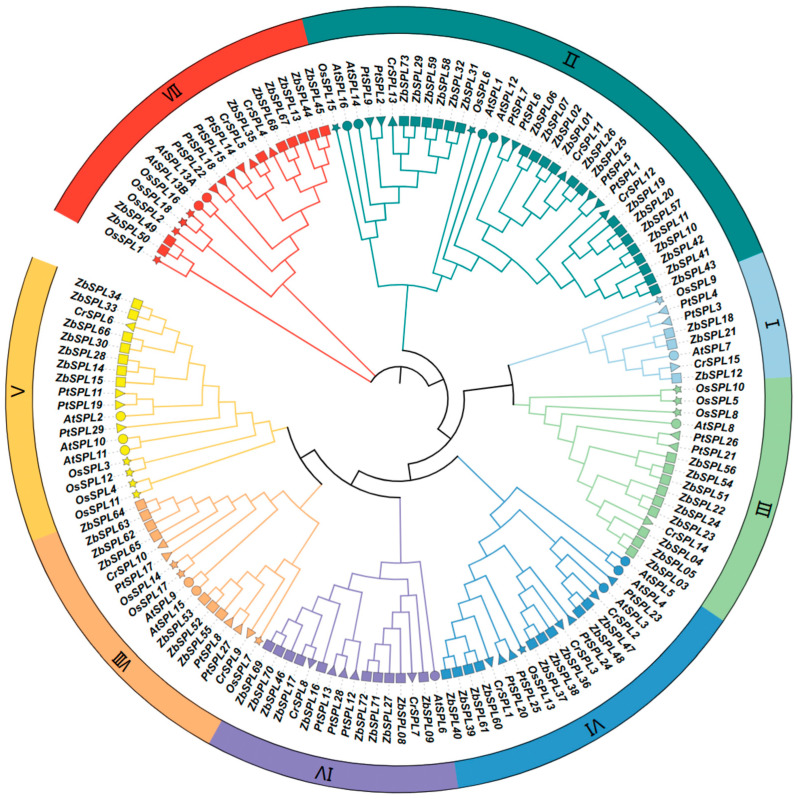
Phylogenetic tree of *SPL* family genes from *Zb*, *At*, *Os*, *Cr*, and *Pt*.

To investigate the evolutionary relationships of the ZbSPLs, an additional 78 SPL proteins were selected for phylogenetic analysis, comprising 17 AtSPLs, 18 OsSPLs, 15 CsSPLs, and 28 PtSPLs from *At*, *Os*, *Citrus reticulata* (*Cr*), and *Populus tomentosa* (*Pt*), respectively. Following the classification method used for AtSPLs, the SPL proteins were categorized into eight groups, with each group containing at least one AtSPL. Subfamily II is the largest, comprising 20 ZbSPLs, followed by subfamilies IV and VI, which contain 9 and 10 members, respectively. Subfamily I is the smallest, with only three members. Proteins that group together in a single branch of the evolutionary tree are likely to share comparable structural traits and potentially have similar biological roles (Figure 4).

### 2.5. Cis-Acting Elements

**Figure 5:**
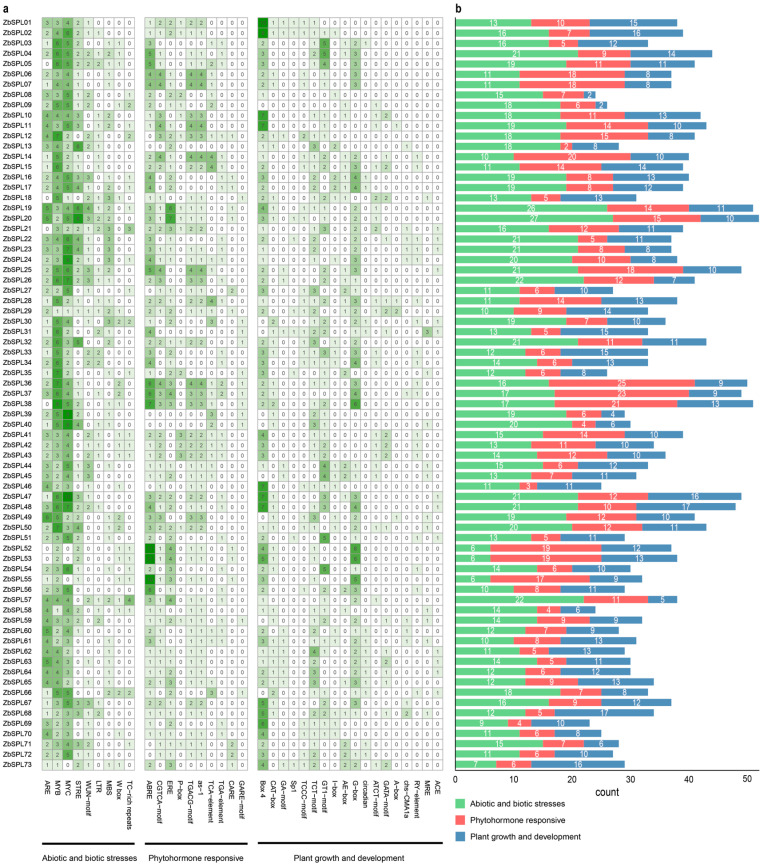
The quantity and arrangement of *cis*-elements in the promoter regions of the *ZbSPL* genes. (**a**) Heat maps showing *cis*-acting components; the number indicates how many *cis*-regulatory elements are present in the promoter region of that *ZbSPL* gene. (**b**) The number of *cis*-reactive element types within each response category for each gene is displayed in the bar chart (according to color).

Using the 2000 bp upstream of the start codon, we examined the promoter *cis*-regulatory element sequences in the *ZbSPL* genes, to elucidate their potential transcriptional regulatory roles. The functions of *ZbSPL* genes can be categorized into three main groups: responses to abiotic and biotic stresses, phytohormone responsiveness, and plant growth and development. The number of elements in all of the genes’ promoters spans from 23 (*ZbSPL69*) to 52 (*ZbSPL20*) (Figure 5b). Of these categories, elements related to plant growth and development are the most prevalent, with 18 distinct types identified (Figure 5a). Almost all of the *ZbSPL* gene promoters contain light-responsive elements. Phytohormone-related response elements, including those responsive to abscisic acid (ABA), methyl jasmonate (MeJA), salicylic acid (SA), auxin, and gibberellin (GA), are also prominent, suggesting that these hormones may regulate *ZbSPL* genes during growth and development in *Zb*. Additionally, nine types of stress-related elements were identified, with antioxidant response elements (ARE) being widely distributed among these *ZbSPL* genes. Thus, each *ZbSPL* gene exhibits a unique composition of regulatory elements. For example, *ZbSPL52*, *ZbSPL53*, and *ZbSPL55* are enriched with abscisic acid response elements (ABREs), while *ZbSPL01* and *ZbSPL02* have a higher number of Box 4 light-responsive elements.

### 2.6. Patterns of Codon Use in the ZbSPL Genes

**Figure 6:**
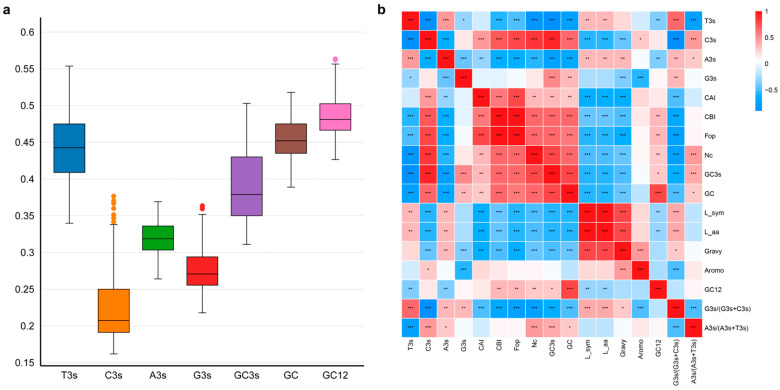
The *ZbSPL* gene family’s codon composition (**a**) and parameter correlation analysis (**b**). In the figure on the left, box shapes of different colors correspond to different parameters below. In the figure on the right, the more stars there are, the higher the correlation. Red represents a highly significant positive correlation, while blue represents a highly significant negative correlation.

**Figure 7:**
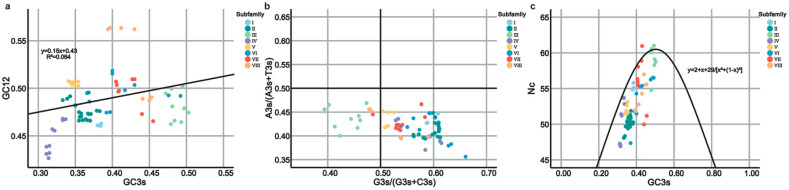
Neutrality plot (**a**), PR2-plot (**b**), and ENC-plot (**c**). The various colors in the upper-right corner indicate the distinct subfamilies to which the *ZbSPL* genes belong.

Utilizing CodonW and EMBOSS, the codon use patterns of the *ZbSPLs* were examined to understand the significant influence of gene evolution and mutations on gene expression levels and functional differentiation. In the *ZbSPLs*, the percentages for each of the four bases in the third position of synonymous codons are 44.10% for T3s, 31.88% for A3s, 22.89% for C3s, and 27.58% for G3s (Figure 6a). The proportion of T3s and A3s is much greater than that of C3s and G3s, indicating that codons in the ZbSPL gene family are more likely to end in an A/T base than a G/C base. Pearson correlation analysis revealed that ENC has a strong positive correlation with GC, GC3s, CAI, CBI, Fop, and C3s, but a significant negative correlation with T3s and A3s (Figure 6b). This implied that the higher the content of A and T in bases of a *ZbSPL*, the stronger its codon preference. Neutral analysis showed that the regression coefficient after data fitting was 0.15, with a low coefficient of determination (R^2^) (0.084), indicating that there is a large difference in the composition preference of the first, second, and third bases of each codon, and that the codon preference has been greatly affected by natural selection (Figure 7a). In Figure 7b, gene loci were primarily found in the lower half of the region, with the regions A3/(A3 + T3) < 0.5 and G3/(G3 + C3) > 0.5 having the largest distributions. According to the findings, the third base use follows the order T > A, G > C, reflecting the preference in selection for this position in the ZbSPL genes. Codon preference is mostly influenced by natural selection. As shown in Figure 7c, gene loci are mainly distributed in the lower side of the standard curve, with some distributed near or on the upper side of the standard curve, indicating that the codon use preference of *ZbSPL* gene is mainly affected by natural selection.

An essential statistic for determining codon preference is RSCU. The most preferred codons are those with RSCU values > 1 and a ∆RSCU ≥ 0.08. The range of optimum codons was found to be between six and 20 for the eight subfamilies. Seven of these codons (TGG, TAG, TTT, GAC, AAT, TTG, and CAG) were found in only a single subfamily. Based on their presence in six subfamilies, the four codons CAA, GAT, TTC, and TGC exhibited the most widespread distribution (Figure S3).

### 2.7. miR156/SPL Module Prediction

**Figure 8:**
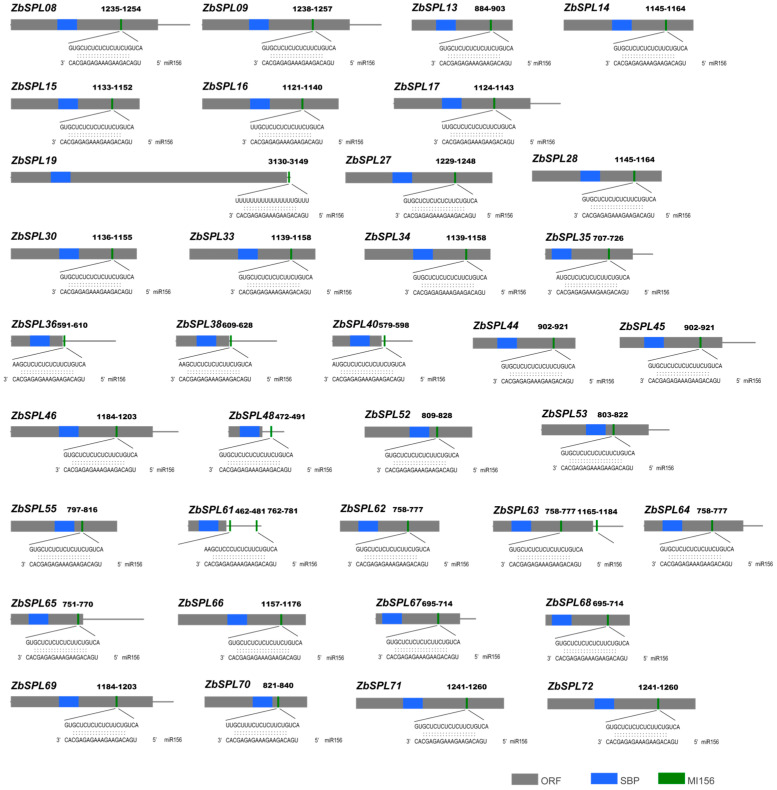
MiR156 predicted target sites on the *ZbSPL* genes. The open reading frame is represented by the gray area, the SBP domain by the blue area, and the complementary sites predicted by miR156 and represented by the green area, with the specific sequences at the bottom and the position information at the top. The gray line segment on the far right is the 3’UTR.

The miR156/*SPL* module is essential for tolerance to abiotic stress, which highlights the importance of identifying *ZbSPL* genes potentially regulated by miR156. A total of 36 *ZbSPL* genes containing target sites for miR156 were identified, representing nearly half of the *ZbSPL* gene family (Figure 8, Table S5). Of these 36 genes, 29 (81%) possess target sites within the coding region, while seven (19%) have target sites within the 3’ UTR. Notably, *ZbSPL63* is the only gene with target sites in both the coding region and the 3’ UTR, whereas *ZbSPL61* is unique in having two target sites within the 3’ UTR.

### 2.8. The Expression Profile of the ZbSPL Genes in Response to Abiotic Stress

**Figure 9:**
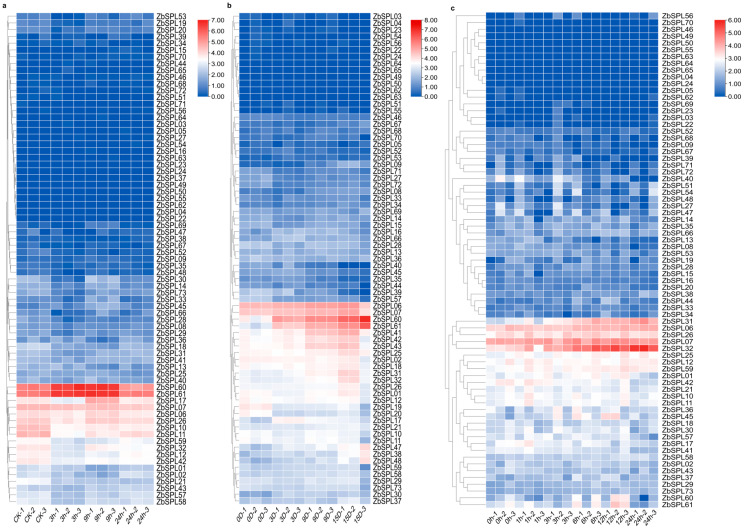
Based on fragments per kilobase of transcript per million mapped reads (FPKM) values, heatmaps of *ZbSPL* expression patterns for salt stress (**a**), drought stress (**b**), and cold stress (**c**) are presented. TBtools was used to visualize the log2 (FPKM) values. The degree of gene expression is shown on the upper-right scale.

To further investigate the functions of the *ZbSPL* gene family under adverse stresses, we analyzed the transcriptional expression patterns of the *ZbSPL* genes using transcriptomic data from *Zb* plants subjected to salt, drought, and cold stress (Figure 9). *ZbSPL06* and *ZbSPL07* exhibited relatively high expression levels under all three abiotic stress conditions, but showed no significant change trends. Under salt stress, *ZbSPL60* and *ZbSPL61* displayed higher expression levels in the control group, which initially increased with treatment and then decreased over time (Figure 9a). When experiencing drought stress, both genes are clearly upregulated (Figure 9b), while no significant changes are observed under cold stress (Figure 9c). *ZbSPL10*, *ZbSPL11*, *ZbSPL12*, *ZbSPL17*, *ZbSPL32*, *ZbSPL42*, and *ZbSPL59* all exhibit a decrease in expression levels during the early stages of salt treatment, followed by a gradual increase over time. During drought stress, their expression remains relatively stable, with only *ZbSPL32* showing significant upregulation under cold stress. *ZbSPL41*, *ZbSPL42*, and *ZbSPL43* are significantly upregulated only under drought stress, with no noticeable changes under salt or cold stress. Finally, *ZbSPL31* shows a distinct upregulation only under cold stress. Overall, the different *ZbSPL* genes exhibit diverse expression responses to salt, drought, and cold stresses, with some genes showing specific regulation under particular stress conditions, while others display a more general response to abiotic stress or stable expression patterns.

**Figure 10:**
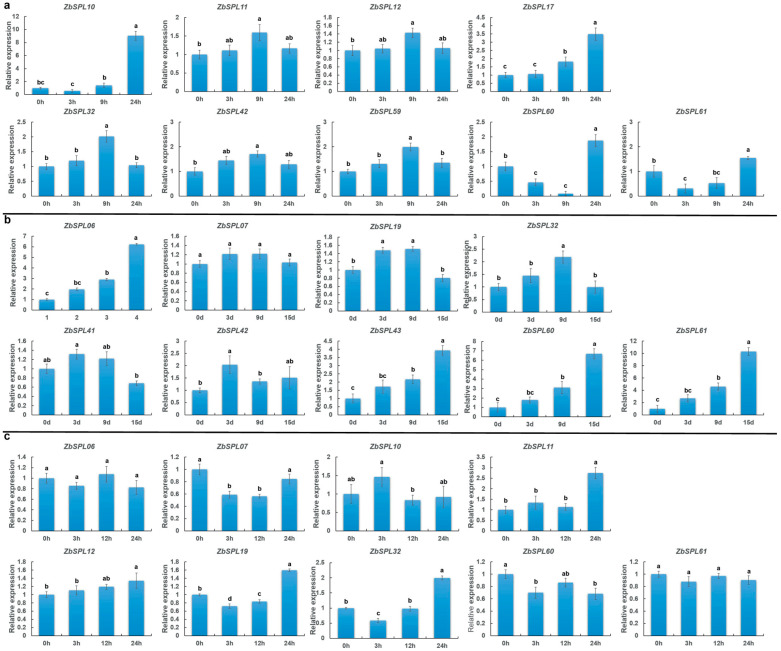
Relative expression levels of 9 *ZbSPL* genes under salt (**a**), drought (**b**), and cold (**c**) stress. Significant variations between various time periods are indicated by distinct letters. LSD tests were used to examine significant differences between groups (*p* < 0.05). The mean ± SD is represented by the data points.

Building on these findings, we further performed combined transcriptomeand *cis*-regulatory element analysis to identify a subset of *ZbSPL* genes and investigate their expression patterns under abiotic stresses, including salt, drought, and cold. Under salt stress, *ZbSPL10* and *ZbSPL17* exhibited similar expression profiles, with *ZbSPL10* showing strong upregulation at 24 h of salt treatment (nearly a 10-fold increase). *ZbSPL11*, *ZbSPL12*, *ZbSPL32*, *ZbSPL42*, and *ZbSPL59* exhibited similar expression patterns, showing significant upregulation at 9 h, followed by downregulation. *ZbSPL60* and *ZbSPL61* were significantly increased at 24 h following salt treatment; however, they were suppressed earlier at 9 h and 3 h, respectively (Figure 10a). *ZbSPL06*, *ZbSPL43*, *ZbSPL60*, and *ZbSPL61* all exhibited notable upregulation following 9 h of drought stress. *ZbSPL07* and *ZbSPL41* displayed a pattern of initial upregulation followed by downregulation; however, these changes were not as pronounced. *ZbSPL42* was significantly upregulated at 3 h, followed by a notable downregulation (Figure 10b). Under cold stress, *ZbSPL07*, *ZbSPL19*, and *ZbSPL32* were strongly and rapidly suppressed early on, but subsequently exhibited gradual induction (Figure 10c).

## 3. Discussion

A set of plant-specific transcription factors known as *SPL* genes, characterized by highly conserved SBP domains, are critical for plant growth, development, and responses to both abiotic and biotic stresses. The acquisition of the whole-genome sequence of *Zb* provides strong support for the comprehensive genome-wide identification of *ZbSPL* genes [30]. In our study, we identified a total of 73 *ZbSPL* family members, surpassing the 8 identified in sugar beet [31], 36 in *Fraxinus mandshurica* [32], 56 in *Vaccinium corymbosum* [33], 22 in *Pisum sativum* [34], and 23 in *Ms* [35], but fewer than the 177 found in cotton (*Gossypium* spp.) [36]. The variation in the number of *SPL* family members across different plant species suggests differential contraction and expansion of the *SPL* gene family during long-term evolutionary processes [37].

In the *Zb* genome, 107 pairs of segmental repeats and 2 pairs of tandem repeats of the *ZbSPL* genes were identified, indicating that segmental repeats are the primary contributors to the expansion of the *ZbSPL* gene family. These findings are consistent with the results observed in the *SPL* gene families of the *Cicer arietinum* and *Carya illinoinensis* genomes, where segmental repeats are the main driving force in the evolutionary process [38,39]. The Ka/Ks ratios for all homologous genes are below 1, suggesting that purifying selection significantly influences the evolution of SPL transcription factors in *Zb*, reflecting a high degree of conservation [40]. Additionally, we compared the repeat patterns in *Zb* with those of six other species. Specifically, we identified 46, 271, 20, 64, 46, and 7 repeat events in comparisons between *Zb* and *At*, *Za*, *Os*, *Cs*, *Csa*, and *Zm*, respectively. The highest collinearity was observed between *Zb* and *Cs*, suggesting their closer evolutionary relationship, which is consistent with the proposal of a recent common ancestor between these two species by Feng et al. [30]. Additionally, the collinearity between *Zb* and dicotyledonous plants exceeds that of with monocotyledonous plants, indicating extensive evolution and duplication of SPL transcription factors following the divergence of monocots and dicots. Phylogenetic analysis of SPL proteins across species reveals that *Zb* is most closely related to *Cs*, which is consistent with the fact that *Zb*, *Cs*, *Pt*, and *At* are all dicotyledonous plants.

Gene structure analysis indicated that members of the *ZbSPL* gene family in subgroup II have the highest number of introns, ranging from nine to eleven, followed by subgroup I with nine introns. In contrast, other subgroups typically have only one or two introns. Early intron theories suggest that eukaryotic ancestors were highly intron-rich, and that intron loss has generally exceeded intron gain throughout evolution [41,42,43,44]. This pattern implies that genes in subgroups V and IV may be more ancient. A previous study indicated that intron loss can contribute to the formation of new genes [45], suggesting that the independent gain and loss of introns may have influenced the expansion of the *ZbSPL* gene family. Different subgroups exhibit distinct motifs and domain characteristics, yet all share highly conserved sequences, particularly motifs 1, 3, and 4. Genes within the same subgroup tend to be more similar; for example, subgroup II consistently has the most motifs. Additionally, some members of subgroup II contain ANKYR domains, which may be involved in protein–protein interactions [46].

Changes in the expression levels of individual genes under particular abiotic stress conditions were validated using qRT-PCR following the RNA-seq study. There were differences in the *Zb* varieties selected, but the healthy annual plants were all selected and treated with the same method. However, comparison of the results with the transcriptome data revealed differences. There are several plausible reasons for these discrepancies, such as the use of different platforms for qRT-PCR and RNA-seq [47], variations in the reference genes chosen for normalization [48], cultivar-specific variability in *Zb* (a different cultivar was used for the RNASeq and qRT-PCR analysis) [49], and the intrinsic complexity of transcriptomic data [50].

Plant growth and development are mostly controlled by *cis*-regulatory elements linked to auxin (IAA), gibberellin (GA), salicylic acid (SA), abscisic acid (ABA), methyl jasmonate (MeJA), and abiotic stress [51,52]. According to our findings, the promoter region of the *ZbSPL* genes were predicted to have a significant number of *cis*-regulatory elements associated with IAA, GA, SA, ABA, MeJA, and abiotic stress. In contrast to *ZbSPL16*, *ZbSPL43*, *ZbSPL60*, and *ZbSPL61*, which were significantly elevated under drought stress, *ZbSPL10* and *ZbSPL17* were highly upregulated under salt stress, according to qRT-PCR data. These results imply that *ZbSPLs* might be crucial in controlling stress responses and hormone-dependent pathways. Conserved in plants, the miR156/*SPL* module is essential for controlling plant development and abiotic stress responses [53,54]. This relationship has been observed by investigating the role of miR156 in regulating *AtSPLs* during cold stress: miR156 was upregulated in the cold, which resulted in downregulation of *AtSPL3* and *AtSPL13*, while *AtSPL9*, another target of miR156, increased in expression and was shown to increase freezing tolerance by upregulating *CBF2* expression [55]. In *Ms* leaves, it has been demonstrated that overexpression or knock-down of *MsSPL13* (which can be achieved by miR156) are more drought-resistant [56]. Additionally, Ms’s salt tolerance is substantially enhanced by the miR156/*SPL4* module, with *MsSPL4OE* lines showing greater salt tolerance [57]. *ZbSPL07*, *ZbSPL19*, and *ZbSPL32* displayed comparable expression patterns under cold stress, being markedly downregulated at 3 h and then markedly increased at 24 h, based on the qRT-PCR results. *ZbSPL60* and *ZbSPL61* also showed a similar pattern of expression when treated with salt. *ZbSPL19* and *ZbSPL61*, targets of miR156, exhibit a suppression-response mode under stress, which may be the result of regulation by miR156.

## 4. Conclusions

The results of this investigation demonstrated there are 73 *ZbSPL* genes in total, which could mostly be categorized into eight subfamilies. Purifying selection has been essential to the formation of the SPL transcription factor family in Zb, with segmental duplication being the primary mechanism for gene family expansion. Synteny analysis revealed the close evolutionary relationship between *Zb* and *Cs*. Numerous regulatory elements linked to plant hormone signaling, light responses, and stress signaling pathways were found in the promoter regions of *ZbSPL* genes. According to the examination of usage patterns, codons of *ZbSPL* gene family members are more likely to end in having A/T bases. The preference for such codons is enhanced with increasing A/T content, suggesting that the use pattern is mostly influenced by natural selection. Moreover, 36 *ZbSPL* genes are anticipated to be possible targets of miR156, suggesting they are likely regulated by this miRNA. Expression profile data show variations in the expression levels of the *ZbSPL* genes under abiotic stress. The qRT-PCR results for the *ZbSPL* genes under different abiotic stress settings suggests that some may be involved in responses to drought and salt stresses. These findings establish a foundation for thoroughly investigating the biological role of *bSPL* genes in *Zb* and offer a fresh viewpoint on their evolution and biological relevance.

## 5. Materials and Methods

### 5.1. Identification and Physicochemical Analysis of ZbSPL Gene Family Members

The genomic data and annotation information for *Zb* were obtained from BioProject ID PRJNA524242 [30]. The amino acid sequences of 16 known SPL proteins from *At* were obtained from the TAIR database (http://www.arabidopsis.org, accessed on 12 July 2024). These *AtSPL* sequences were used as queries in BLAST searches against the *Zb* genome to identify all candidate *ZbSPL* genes. To exclude non-SPL domain sequences and eliminate redundancy, annotations were cross-referenced using the CDD database (http://www.ncbi.nlm.nih.gov/Structure/cdd/wrpsb.cgi, accessed on 12 July 2024) and the Pfam database (http://pfam.xfam.org/, accessed on 12 July 2024). The physicochemical properties of the *ZbSPL* gene family members were predicted using the ExPasy online tool (https://web.expasy.org/, accessed on 12 July 2024). Subcellular localization predictions were performed using the WoLF PSORT tool (https://wolfpsort.hgc.jp/, accessed on 12 July 2024).

### 5.2. Chromosome Positioning and Gene Replication

The physical position information of *ZbSPL* genes in *Zb* was extracted using the TBtools v2.149 software [58], mapped onto the chromosomes, and then visualized. Gene duplication events were analyzed using the MCScanX toolkit with default parameters to conduct a synteny analysis of the *ZbSPL* genes, and the results were also processed using TBtools. The Ka/Ks ratio was computed to evaluate the selection pressure on the duplicated genes.

### 5.3. Gene Structure, Conserved Motifs, and Conserved Binding Domains

Multiple sequence alignment of the ZbSPL proteins was conducted using MEGA7 [59]. The full-length protein sequences of the ZbSPL family’ members were examined to determine the top 20 conserved motifs using the MEME web tool (https://meme-suite.org/meme/tools/MEME, accessed on 12 July 2024).

Conserved domains in the ZbSPL proteins were investigated using the NCBI Batch CD-Search tool. The visualization of conserved domains, motifs, and intron/exon structures of the ZbSPL proteins was performed using TBtools.

### 5.4. Phylogenetic Analysis of ZbSPLs

*At*, *Os*, *Cr*, and *Pt* SPL protein sequences are provided in Table S4. The protein sequences were aligned using MEGA 7.0 ’s Muscle function. The optimal amino acid substitution model was selected using the maximum likelihood (ML) approach, and tree reliability was assessed through 1000 bootstrap replicates. The iTOL online tool (https://itol.embl.de/, accessed on 18 July 2024) was used to process and illustrate the final phylogenetic relationships.

### 5.5. Examination of Codon Usage Bias

Codon composition (GC3s, T3s, C3s, A3s, G3s, GC, and GC12), relative synonymous codon use (RSCU), optimal codon (Fop), effective codon number (ENC), codon adaptation index (CAI), and codon bias index (CBI) were all calculated using the CodonW software. The RSCUs and ΔRSCUs of the eight subgroups were calculated using the EMBOSS website (https://www.bioinformatics.nl/cgi-bin/emboss/cusp, accessed on 24 July 2024). The most preferred codons are those with a high frequency (RSCU > 1) and high expression (ΔRSCU ≥ 0.08). SPSS (Version 26) was utilized for the scatter plot and correlation analysis. ChiPlot (https://www.chiplot.online/, accessed on 24 July 2024) was used to generate box plots and upset plots.

### 5.6. Cis-Acting Elements and miR156 Prediction

Potential *cis*-acting elements within the 2000 bp upstream promoter regions of the *ZbSPL* genes were predicted using the PlantCare website (https://bioinformatics.psb.ugent.be/webtools/plantcare/html/, accessed on 18 July 2024). Data extraction and organization were performed using the TBtools v2.149 software, utilizing genomic data and annotation information. Based on the previous classification, the results from using R’s ggplot function are shown [60]. The psRNATarget tool (https://www.zhaolab.org/psRNATarget/, accessed on 18 July 2024) was employed to search for complementary sequences of miR156 in the coding and 3’ UTR regions of the *ZbSPL* genes.

### 5.7. Transcriptome Analysis

RNA-seq data for *Zb* under drought (PRJNA784034), cold stress (PRJNA597398), and salt stress (PRJNA1107841) conditions were retrieved from the NCBI SRA database. Initially, SRA files were converted to FASTQ format using the fastdump tool of the SRA Toolkit [61]. The quality of the raw sequencing data was assessed using FastQC [62]. Adapter sequences and low-quality reads were subsequently removed using Trimmomatic [63] to produced clean data. The clean data were then aligned to the *Zb* genome using Hisat2 [64]. The unified FPKM value was expressed after the gene expression value was calculated using String-Tie [65]. Heatmaps and clustering of log2 (FPKM) values for the *ZbSPL* genes were generated using ChiPlot.

### 5.8. Plant Materials and qRT-PCR

One-year-old *Zb* plants, “Fengxiandahongpao”, were raised in a greenhouse at 25 °C, 60–70% humidity, and a 16/8 h light/dark cycle. After treating plants to salt stress with a 250 mmol/L NaCl solution, leaf samples were collected at 0 h, 3 h, 9 h, and 24 h. To obtain leaf samples for drought stress, irrigation was stopped, and tissue was collected at 0 days (0 d), 6 days (6 d), 9 days (9 d), and 15 days (15 d). Cold stress was implemented using a growth chamber set at 4 °C, and leaf samples were taken at 0 h, 3 h, 12 h, and 24 h. In almost all cases, these methods are based on those used in other RNA-seq studies I quantified. Three biological replicates were cultivated for each time point, with each replicate consisting of three independent seedlings. Samples were immediately frozen in liquid nitrogen and stored at −80 °C until further use in experiments.

RNA was extracted using the FastPure Plant Total RNA Isolation Kit (Vazyme Biotech Co., Ltd., Nanjing, China), and RNA concentration and purity were assessed. cDNA was synthesized using the HiScript II Q RT SuperMix (Vazyme Biotech Co., Ltd., Nanjing, China) for qRT-PCR. Primers were designed using Primer 5.0 software (Table S6) and validated for specificity using the TBtools v2.149 software. The *Zb* actin gene, *ZbActin*, was used as a reference gene [66]. qRT-PCR analysis of the *ZbSPL* genes was performed using the 2×ChamQ Universal SYBR qPCR Master Mix (Vazyme Biotech Co., Ltd., Nanjing, China), with at least three biological replicates and three technical replicates. The 10 µL reaction mixture consisted of 0.5 µL of cDNA template, 0.2 µL of each primer (upstream and downstream), 5 µL of SYBR MIX, and 4.1 µL of RNase-free water. The PCR conditions were as follows: 95 °C for 30 s; followed by 40 cycles of 95 °C for 10 s, 60 °C for 30 s; 65 °C for 5 s; and a final incubation 95 °C for 5 s to obtain a melt curve. Relative quantification was conducted using the 2^(−ΔΔCt)^ method [67]. Statistical analysis was performed with SPSS software (Version 26), with one-way ANOVA followed by the least significant difference (LSD) test. Gene expression levels were visualized using histograms generated in Excel 2021.

## Data Availability

Data sharing is available upon request.

## Supplementary Materials

The following supporting information can be downloaded at https://www.mdpi.com/article/10.3390/plants14040520/s1, Figure S1. Phylogenetic tree, motif patterns, gene structures, and conserved domain of ZbSPL proteins. (**a**) The phylogenetic tree was constructed using the full-length sequences of the ZbSPL proteins with 1000 replicates on each node. (**b**) The amino acid motifs (numbered 1–20) in the ZbSPL proteins are displayed in 20 colored boxes, and the black lines indicate amino acid length. (**c**) Conserved domains for the ZbSPL members: SBP domains, indicated by green boxes; and ANKYR domains, represented by yellow boxes. (**d**) Gene structure of *ZbSPL* members: untranslated region (UTR), represented by green boxes and coding sequence (CDS), represented by yellow boxes. Figure S2. Alignment of the SBP domain in ZbSPL proteins. Sequence logo for the SBP-box in the ZbSPLs. The overall height of each stack indicates the degree of conservation of the corresponding residue site, while the height of the characters in each stack represents the relative frequency. Shown at the bottom are two conserved zinc-binding sites (Zn1 and Zn2) and a nuclear localization signal (NLS). Figure S3. Clustering of the relative synonymous codon usage in the gene of the eight subfamilies. Table S1. Gene names and locus information for the SPL proteins in *Zb*. Table S2. Analysis of the physical and chemical properties and prediction of the ZbSPLs of their cell localization in *Zb*. Table S3. Ka/Ks ratio of *ZbSPL* genes in clades I–VIII. Table S4. Sequence features of *AtSPLs* in *A. thaliana*, *O. sativa*, *C. reticulata*, and *Populus tomentosa*. Table S5. *ZbSPLs* targeted by miR156. Table S6. Primers for real-time quantitative PCR.
