# Supplementary material for: Identification and Characterization of SQUAMOSA Promoter Binding Protein-like Transcription Factor Family Members in Zanthoxylum bungeanum and Their Expression Profiles in Response to Abiotic Stresses
Source: Plants (Basel). 2025 Feb 8;14(4):520. doi: 10.3390/plants14040520 (PMC11859874; doi:10.3390/plants14040520)
Supplement: Supplementary file 1 [file plants-14-00520-s001.zip › Supporting Information.pdf]

Identification and Characterization of SQUAMOSA Promoter Binding Protein-like Transcription Factor Family Members in *Zanthoxylum bungeanum* and Their Expression Profiles in Response to Abiotic Stresses

Shengshu Wang [1,2,†], Weiming Hu [2,†], Xueli Zhang [1], Yulin Liu [1,*] and Fen Liu [2,*]

[1] College of Forestry, Northwest A&F University, Yangling 712100, China; wss24616@163.com (S.W.); zxl980505@nwafu.edu.cn (X.Z.)
[2] Lushan Botanical Garden, Jiangxi Province and Chinese Academy of Sciences, Jiujiang 332900, China; huwm@lsbg.cn
* Correspondence: liuyulin@nwafu.edu.cn (Y.L.); liuf@lsbg.cn (F.L.)
† These authors contributed equally to this work.

The following Supporting Information is available for this article:

Supporting Information

Figure S1. Phylogenetic tree, motif pattern, gene structure, and conserved domain of ZbSPL proteins. (a) The phylogenetic tree was constructed using the full-length sequences of ZbSPL proteins with 1000 replicates on each node. (b) The amino acid motifs (numbered 1-20) in ZbSPL proteins are displayed in 20 colored boxes, and black lines indicate amino acid length. (c) Conserved domains for ZbSPL members: SBP domains, indicated by green boxes; and ANKYR domains, represented by yellow boxes. (d) Gene structure of *ZbSPL* members: UTR, untranslated region; represented by green boxes; CDS, and coding sequence; represented by yellow boxes.

Figure S2. Alignment of the SBP domain in ZbSPL proteins. Sequence logo for ZbSPLs' SBP-box. The overall height of each stack indicates the degree of conservation of the corresponding residue site, while the height of the characters in each stack represents the relative frequency. At the bottom, two conserved zinc-binding sites (Zn1 and Zn2) and a nuclear localization signal (NLS) are shown.

Figure S3. Clustering of the relative synonymous codon usage in gene families of eight subfamilies.

Table S1. Gene names and locus information for the SPL proteins in Zb.

Table S2. Analysis of physical and chemical properties and prediction of cell localization in Zb.

Table S3. Ka/Ks ratio of *ZbSPL* genes in clades I-VIII.

Table S4. Sequence features of *AtSPLs* in *A. thaliana*, *O. sativa*, *C. reticulata*, and *Populus tomentosa*.

Table S5. ZbSPLs targeted by miR156.

Table S6. Primers for real-time quantitative PCR.

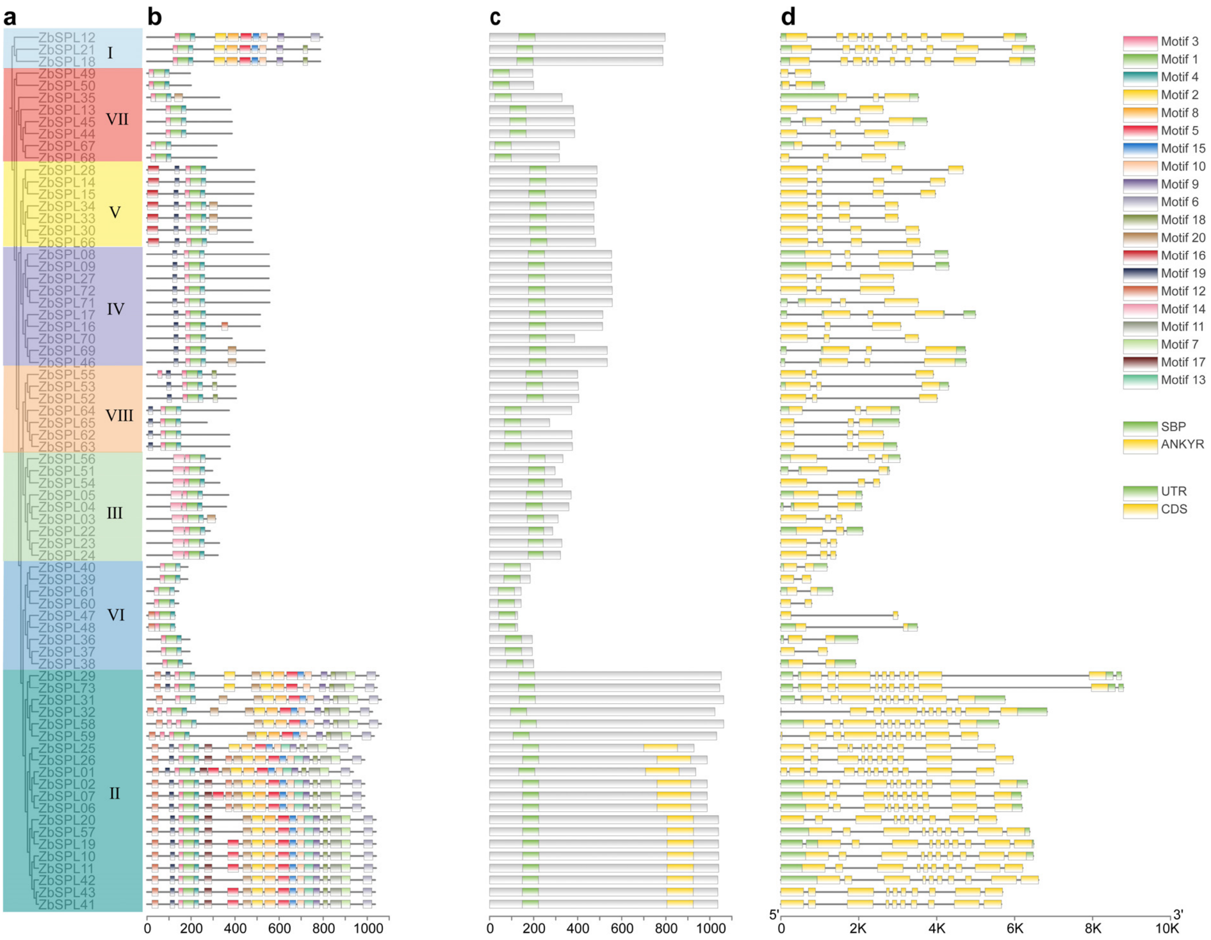

Figure S1. Phylogenetic tree, motif pattern, gene structure, and conserved domain of ZbSPL proteins. (a) The phylogenetic tree was constructed using the full-length sequences of ZbSPL proteins with 1000 replicates on each node. (b) The amino acid motifs (numbered 1-20) in ZbSPL proteins are displayed in 20 colored boxes, and black lines indicate amino acid length. (c) Conserved domains for ZbSPL members: SBP domains, indicated by green boxes; and ANKYR domains, represented by yellow boxes. (d) Gene structure of *ZbSPL* members: UTR, untranslated region; represented by green boxes; CDS, and coding sequence; represented by yellow boxes.

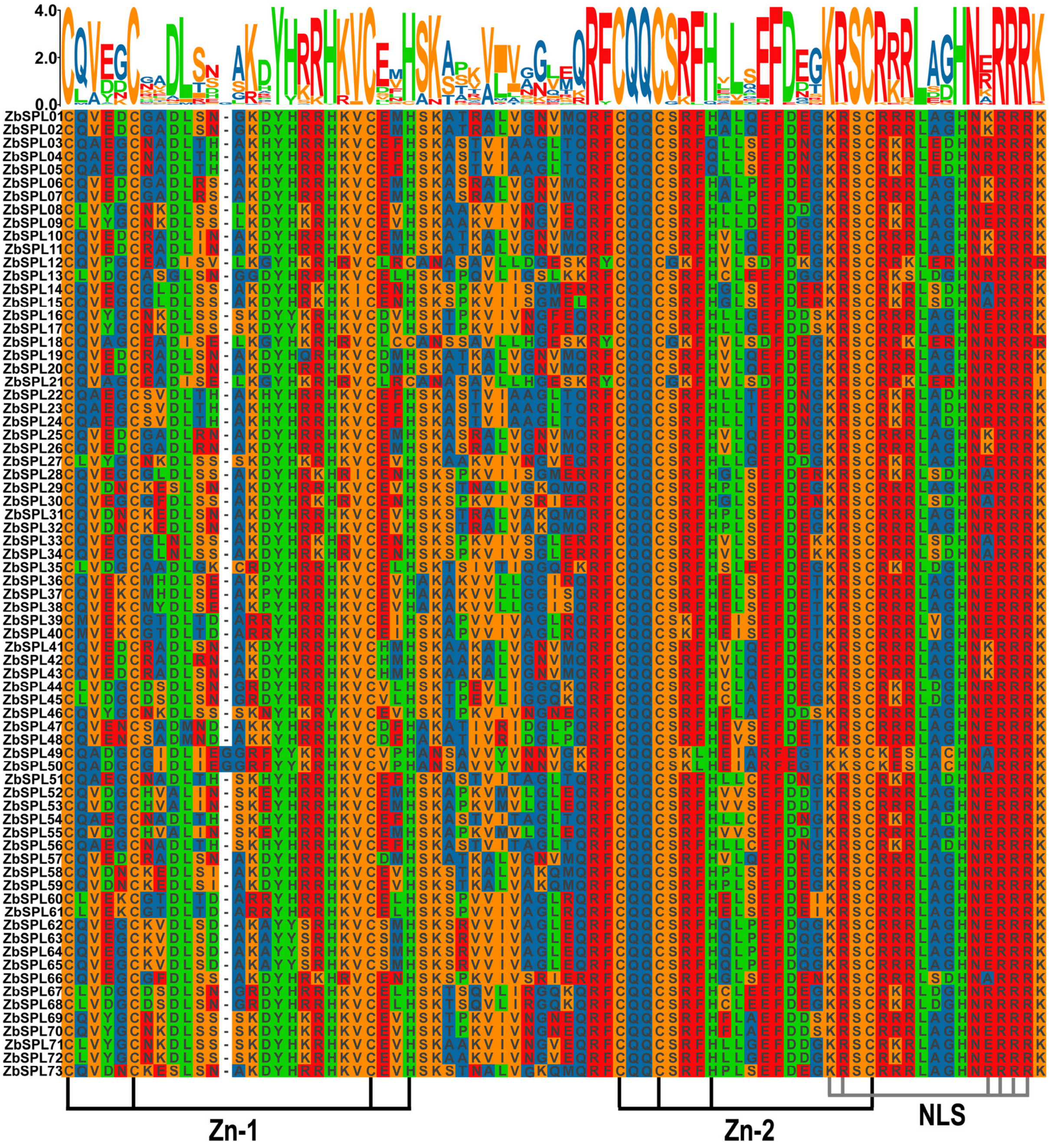

Figure S2. Alignment of the SBP domain in ZbSPL proteins. Sequence logo for ZbSPLs' SBP-box. The overall height of each stack indicates the degree of conservation of the corresponding residue site, while the height of the characters in each stack represents the relative frequency. At the bottom, two conserved zinc-binding sites (Zn1 and Zn2) and a nuclear localization signal (NLS) are shown.

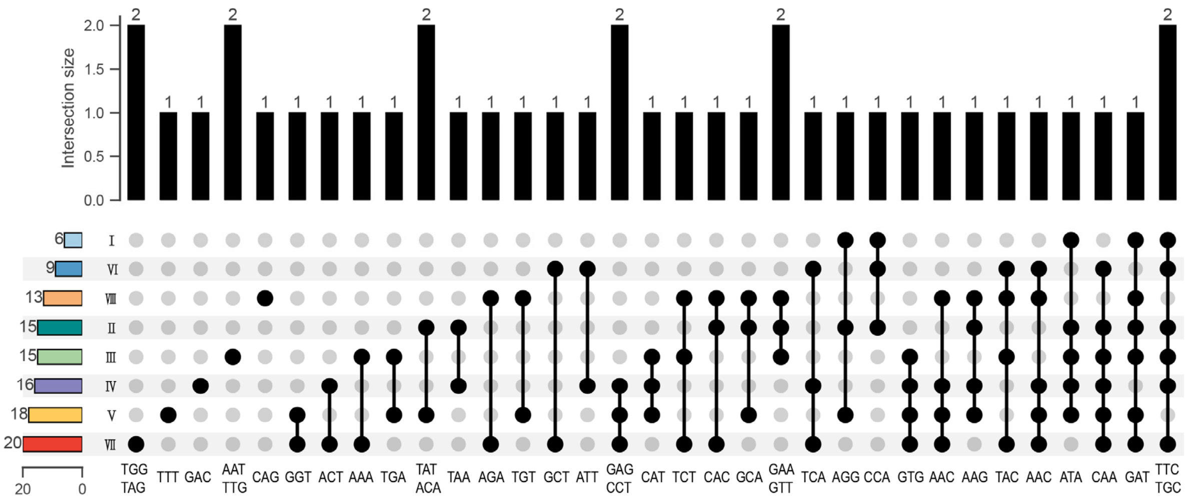

Figure S3. Clustering of the relative synonymous codon usage in gene families of eight subfamilies.
